# Serum Amyloid A as an Inflammatory Biomarker in Captive Japanese Sika Deer (*Cervus nippon*): Reference Intervals and Correlations with Hematological and Serum Biochemical Parameters

**DOI:** 10.3390/ani16203160

**Published:** 2026-10-09

**Authors:** Ayaka Ito, Hiroko Sakurai, Fumie Maetani, Takayuki Asai, Masahiro Yasuda, Osamu Yamato

**Affiliations:** 1 Laboratory of Veterinary Anatomy, Interdisciplinary Graduate School of Medicine and Veterinary Medicine, University of Miyazaki, Miyazaki 889-2192, Japan; 2 Kagoshima City Hirakawa Zoological Park, Kagoshima 891-0133, Japan; 3 Joint Graduate School of Veterinary Medicine, Kagoshima University, Kagoshima 890-0065, Japan; 4 Laboratory of Veterinary Anatomy, Department of Veterinary Sciences, Faculty of Agriculture, University of Miyazaki, Miyazaki 889-2192, Japan; 5 Joint Faculty of Veterinary Medicine, Kagoshima University, Kagoshima 890-0065, Japan

**Keywords:** serum amyloid A, Japanese sika deer, *Cervus nippon*, acute phase protein, inflammation, reference interval, VET-SAA, LZ-SAA, animal health, biomarker

## Abstract

Serum amyloid A (SAA) is a major acute phase protein that increases rapidly during inflammation, infection, and tissue injury, but data on SAA in Japanese sika deer (*Cervus nippon*) remain limited. SAA concentrations were measured in 128 serum samples from 84 captive Japanese sika deer (Mageshima sika deer, n = 26; Formosan sika deer, n = 44; mixed-subspecies individuals, n = 14) at Kagoshima City Hirakawa Zoological Park, Japan, using two latex agglutination turbidimetric immunoassays, VET-SAA, and LZ-SAA. Reference intervals were established, and Spearman rank correlations were evaluated between SAA and hematological and serum biochemical parameters. SAA concentrations were also compared between healthy (n = 30) and diseased (n = 95) groups, and receiver operating characteristic (ROC) analysis determined optimal cutoff values. Reference intervals for VET-SAA and LZ-SAA were 0.00–6.73 mg/L and 0.00–0.96 mg/L, respectively. Both assays correlated significantly with total protein, globulin, albumin, the albumin-to-globulin ratio, band and segmented neutrophils, lymphocytes, and eosinophils. SAA concentrations were also significantly higher in diseased than healthy deer (*p* < 0.001). The area under the ROC curve was 0.972 for VET-SAA and 0.959 for LZ-SAA. These findings suggest that SAA reflects inflammatory status in Japanese sika deer and may serve as a useful supplementary biomarker for inflammatory assessment in this species.

## Simple Summary

Serum amyloid A (SAA) is a protein found in the blood that rises quickly when an animal is sick, injured, or fighting an infection, making it a useful sign of overall health. Little is known, however, about typical SAA levels in Japanese sika deer, a species commonly kept in Japanese zoos. In this study, blood samples from 84 sika deer housed at a zoo in Kagoshima, Japan, were tested for SAA using two different commercially available test kits, called VET-SAA and LZ-SAA. We established normal reference intervals for SAA in this species and examined how SAA levels related to other blood measurements, such as protein levels and white blood cell counts, that are also used to check for inflammation. We also compared SAA levels between deer that appeared healthy and those with a diagnosed illness. Deer with illness had much higher SAA levels than healthy deer, and SAA levels were closely linked to several other markers commonly used to detect inflammation. These results show that measuring SAA can help veterinarians and zookeepers detect inflammation and monitor the health of Japanese sika deer in captivity, offering a useful additional tool alongside standard blood tests for the care and management of this species in zoos and other captive settings.

## 1. Introduction

The acute phase response is a non-specific systemic reaction to inflammation, infection, and tissue injury, characterized by hepatic production of acute phase proteins (APPs) under stimulation by pro-inflammatory cytokines. Changes in APP concentrations in blood reflect the presence and activity of inflammation and are widely used as diagnostic and monitoring tools in veterinary medicine [1,2]. Serum amyloid A (SAA) is a major APP that is synthesized primarily by hepatocytes and circulates in association with high-density lipoprotein during the acute phase response [1]; it increases rapidly in response to various inflammatory conditions, including infection, tissue damage, and trauma, across a wide range of domestic and non-domestic animal species [3,4].

SAA has been applied as an inflammatory biomarker across many species. In cattle, it has been used to evaluate infectious and periparturient diseases [5,6,7,8], and in horses, for the diagnosis and treatment monitoring of acute inflammatory conditions [9,10]. In small ruminants, SAA has similarly been reported as a useful indicator of inflammatory and infectious disease [11,12]. In dogs and cats, SAA has been reported to be a useful indicator of inflammatory disease [13,14,15]. More recently, SAA measurement has been explored in wild and captive animals such as cetaceans and elephants, where it may detect inflammatory conditions that are difficult to assess by conventional hematological methods alone [16,17,18,19]; such minimally invasive blood-based biomarkers are particularly advantageous in these species, as they reduce the need for repeated physical or chemical restraint associated with more invasive diagnostic procedures.

Reports on SAA in cervids remain limited, although its potential utility has been demonstrated. In white-tailed deer (*Odocoileus virginianus*), reference intervals for SAA have been established, and elevated SAA concentrations have been confirmed in clinically abnormal individuals; SAA has also been considered useful in combination with conventional parameters such as white blood cell (WBC) count and albumin/globulin (A/G) ratio for evaluating inflammatory status [20]. Furthermore, the utility of SAA measurement has been explored in other cervids, including caribou (*Rangifer tarandus granti*) [21]. Nevertheless, reports in cervids remain scarce, and species-specific reference intervals and characteristics as an inflammatory marker have not been fully elucidated. Comparative studies further indicate that the amino-terminal region of SAA, the primary epitope target of most commercial immunoassays, is not fully conserved across species, providing a structural basis for the differing degrees of cross-reactivity observed when species-specific and heterologous SAA assay reagents are applied outside their intended target species [22].

Japanese sika deer (*Cervus nippon*) are widely distributed across East Asia and are frequently encountered in veterinary practice in Japan, including individuals maintained in zoological parks and wildlife rehabilitation facilities. However, reports on SAA in Japanese sika deer are scarce, and reference intervals have not been established. The relationships between SAA and conventional hematological and serum biochemical parameters in this species also remain uninvestigated, leaving the clinical applicability of SAA unclear. As a prey species, sika deer, like many other cervids, tend to mask overt clinical signs of illness [23], making early and objective detection of inflammation particularly valuable for their management in zoological and rehabilitation settings; a validated, minimally invasive blood biomarker such as SAA could therefore support earlier clinical decision-making than reliance on behavioral observation alone.

The objectives of this study were to establish reference intervals for SAA in captive Japanese sika deer using two commercial assay systems, to evaluate correlations between SAA concentrations and hematological and serum biochemical parameters, and to compare SAA concentrations between healthy and diseased individuals, thereby assessing the potential utility of SAA as an inflammatory biomarker in this species.

## 2. Materials and Methods

### 2.1. Animals

A total of 128 serum samples were obtained from 84 captive Japanese sika deer (*Cervus nippon*) maintained at Kagoshima City Hirakawa Zoological Park, Kagoshima, Japan, between December 2008 and October 2020. The study population comprised Mageshima sika deer (n = 26), Formosan sika deer (Hanajika; n = 44), and mixed-subspecies individuals tentatively classified as hybrids with Honshu or Kyushu sika deer subspecies (n = 14) [24,25]. Some individuals were sampled at multiple time points (maximum 8 times per individual).

### 2.2. Experimental Design and Blood Collection Procedure

This variation in sampling frequency was not based on a predetermined study design but reflected the opportunistic use of residual serum from blood samples collected during routine veterinary care; individuals under more frequent clinical monitoring therefore contributed proportionally more samples. Blood was collected primarily by jugular venipuncture as part of standard veterinary procedures; the exact collection volume was not systematically recorded for each sample, as collection was performed for clinical purposes rather than for this study. Blood collection was performed in isolated stalls within the animal housing area at the Zoological Park; although enclosed, these stalls have barred walls exposed to outdoor air. In rare cases involving severely ill individuals, sampling was performed under general anesthesia at the Zoological Park’s on-site animal hospital, but this was uncommon.

### 2.3. Sample Processing and Ethical Approval

Residual serum samples originally collected for clinical examination or routine health check-ups were used. Serum was separated after collection and stored frozen until analysis; most samples were stored at −80 °C, with some stored at −20 °C. Some blood collections were part of scheduled health monitoring in which potential research use of the residual serum was also taken into consideration when the schedule was planned; however, all sample collection remained within the scope of clinically indicated procedures, and no additional invasive procedures were performed solely for the purposes of this study. The use of these residual samples for research purposes was approved by the director and staff of Kagoshima City Hirakawa Zoological Park at a staff meeting. Kagoshima City Hirakawa Zoological Park is a member of the Japanese Association of Zoos and Aquariums (JAZA), the Japan branch of the World Association of Zoos and Aquariums (WAZA), and animal welfare at the facility is managed in accordance with JAZA/WAZA animal welfare standards. Of the 84 individuals, sex was recorded for all animals (male, n = 41; female, n = 43). Birth date was known, either from documented records or estimated from the date of tagging at capture, for 36 individuals, whose age at the time of sampling ranged from 0 to 12 years (median, 3 years); birth date was unknown for the remaining individuals owing to incomplete historical records, as this study included animals sampled over a long-term period (2008–2020).

### 2.4. SAA Measurement

Serum SAA concentrations were measured using two commercial latex agglutination turbidimetric immunoassay (LATIA) systems: VET-SAA and LZ-SAA (Eiken Chemical Co., Ltd., Tokyo, Japan), both analyzed on an automated biochemistry analyzer (Pentra C200; HORIBA ABX SAS, Montpellier, France). Measurements and calibration were performed according to the manufacturer’s instructions. Dilution linearity was assessed using serial two-fold dilutions (1:1 to 1:256) of three high-concentration serum samples. VET-SAA showed a proportional decrease in measured concentration with increasing dilution across the tested range, with linear regression of the observed against expected concentrations (pooled across the three samples, n = 27) yielding a slope of 0.975 (95% CI, 0.946–1.005), an intercept of 6.48 mg/L (95% CI, 3.90–9.06), and R^2^ = 0.995. In contrast, LZ-SAA did not show proportional dilution recovery across most of the tested range (pooled R^2^ = 0.71 across the full dilution series), with measured values remaining disproportionately elevated relative to the expected concentration until high dilution factors (≥1:32), beyond which values declined sharply toward the detection floor (see Section 4.1 for further discussion of the analytical basis for this reduced sensitivity). Because LZ-SAA concentrations across the full sample set (n = 128) included a substantial proportion of non-detected values (LZ-SAA = 0.00 mg/L in 25/128 samples), whereas VET-SAA was detected in all samples (minimum, 0.89 mg/L), the detection limit of LZ-SAA was estimated using the hit-rate method, in which the probability of LZ-SAA detection (0.00 mg/L vs. >0.00 mg/L) was modeled as a function of the corresponding VET-SAA concentration (which showed dilution linearity, unlike LZ-SAA) using logistic regression, following an approach conceptually similar to CLSI EP17 [26]. This analysis indicated that the VET-SAA concentration corresponding to 50% and 95% probability of LZ-SAA detection was 4.14 mg/L and 6.67 mg/L, respectively, providing an indirect estimate of the LZ-SAA detection limit expressed in terms of the co-measured VET-SAA concentration. Because VET-SAA was detected in all 128 samples with no non-detected values, an equivalent estimate of the VET-SAA detection limit could not be derived from this dataset. Intra-assay precision (coefficient of variation, CV) was determined by measuring high-, medium-, and low-concentration serum samples in 10–12 replicates. Intra-assay CVs for VET-SAA were 3.15% (mean, 96.3 mg/L; n = 12), 4.45% (mean, 24.24 mg/L; n = 10), and 9.89% (mean, 4.46 mg/L; n = 11) at high, medium, and low concentrations, respectively, all within generally accepted precision limits (CV < 10–20%). For LZ-SAA, intra-assay CVs were 1.42% (mean, 81.48 mg/L; n = 12) and 1.74% (mean, 19.78 mg/L; n = 10) at high and medium concentrations, respectively, but markedly increased to 51.06% (mean, 0.25 mg/L; n = 11) at the low concentration, exceeding acceptable precision limits; this large imprecision at low concentrations is consistent with this concentration range approaching the LZ-SAA detection limit estimated above.

The healthy group was strictly defined as individuals with no apparent clinical abnormalities at the time of blood collection and no abnormal findings in any hematological or serum biochemical parameter (including WBC, differential counts, total protein (TP), albumin (Alb), globulin (Glb), and A/G ratio) (n = 30); these samples were also used to establish the reference intervals. Samples collected for routine health check-ups, periodic monitoring, hoof trimming, post-discharge follow-up, post-fracture follow-up, and implant insertion that nonetheless showed an abnormality in any hematological or serum biochemical parameter were not included in the healthy group and were instead classified as diseased. The diseased group therefore comprised all remaining individuals, including those with overt clinical abnormalities such as trauma, abdominal perforation, rumenal perforation, abscess, suspected mastitis, weakness, loss of appetite, neurological signs, hypothermia, hypoglycemia, dehydration, hematochezia, respiratory distress, vomiting, injuries from fighting, and post-mortem sampling, together with the samples described above that showed laboratory abnormalities despite an unremarkable clinical presentation. Of the 128 serum samples collected, two were excluded from analysis because the measured SAA concentrations were considered unreliable owing to dilution-related assay issues, and one further sample was excluded as a likely duplicate measurement of another sample from the same individual and date, leaving 125 samples (30 healthy, 95 diseased) for the healthy versus diseased comparison, ROC analysis, and correlation analyses. Because health status was evaluated per sample (i.e., per blood collection) rather than per individual, an individual whose clinical and laboratory status changed between two sampling occasions could contribute one sample to the healthy group and a separate sample to the diseased group; this occurred for 9 of the 84 individuals in this study. This cross-group overlap, together with the repeated sampling of individuals within each group, is explicitly accounted for in the statistical analysis described in Section 2.6.

### 2.5. Hematological and Serum Biochemical Analyses

Hematological parameters included total WBC count, WBC differential, and platelet count (PLT). Complete blood counts were performed using the pochi-100iV analyzer (ARKRAY, Inc., Kyoto, Japan). Differential counts were performed manually on blood smears stained with Diff-Quik, with 100 leukocytes counted per sample under light microscopy; proportions of band neutrophils, segmented neutrophils, lymphocytes, monocytes, and eosinophils were determined.

Serum biochemical parameters included TP, Alb, Glb, and A/G ratio, measured using the DRI-CHEM 7000V analyzer (FUJIFILM Corporation, Tokyo, Japan). Glb concentration was calculated by subtracting Alb from TP; the A/G ratio was calculated by dividing Alb by Glb.

### 2.6. Statistical Analysis

Because some individuals were sampled repeatedly (up to 8 times), the assumption of statistical independence required for conventional Spearman correlation and Mann–Whitney U tests were not fully met. To address this, repeated measures correlation (rmcorr), which estimates the common within-individual association between two variables while accounting for non-independence of repeated observations from the same individual, was used to evaluate relationships between SAA concentrations (log-transformed) and hematological and serum biochemical parameters, with individual identity specified as the repeated-measures factor [27]. SAA concentrations (log-transformed) between the healthy and diseased groups were compared using a linear mixed-effects model with group as a fixed effect and individual identity as a random intercept, to account for the repeated sampling of some individuals. This individual-level random-intercept structure also appropriately accounts for the 9 individuals that contributed a sample to both the healthy and diseased groups (once while healthy and once while diseased), because the model treats an individual’s samples as correlated observations regardless of which group they fall into, rather than assuming full independence between groups. As a sensitivity analysis, the original Spearman correlation and Mann–Whitney U test were repeated using a single (first chronological) sample per individual (n = 84); the direction and statistical significance of the principal associations were consistent with the repeated-measures-adjusted analyses, supporting the robustness of the findings. ROC analysis was performed to evaluate the diagnostic ability of each assay to discriminate between healthy and diseased individuals, and the area under the ROC curve (AUC) with 95% confidence intervals (CI) was calculated using the bootstrap method (2000 iterations). To account for the non-independence of repeated samples from the same individual, a cluster bootstrap was additionally performed, resampling at the individual level (rather than the sample level) in each of the 2000 iterations. The optimal cutoff value was determined as the point maximizing the Youden index (sensitivity + specificity − 1) [28]. Statistical significance was set at *p* < 0.05. As this was an exploratory study, correction for multiple comparisons was not applied. All statistical analyses were performed using Python (version 3.11; SciPy version 1.13, scikit-learn version 1.5, statsmodels version 0.14, and Pingouin version 0.5 libraries). The rmcorr-adjusted correlations are presented alongside the original Spearman correlations for comparison in the Results section.

## 3. Results

### 3.1. Comparison of SAA Concentrations Between Healthy and Diseased Groups

**Figure 1:**
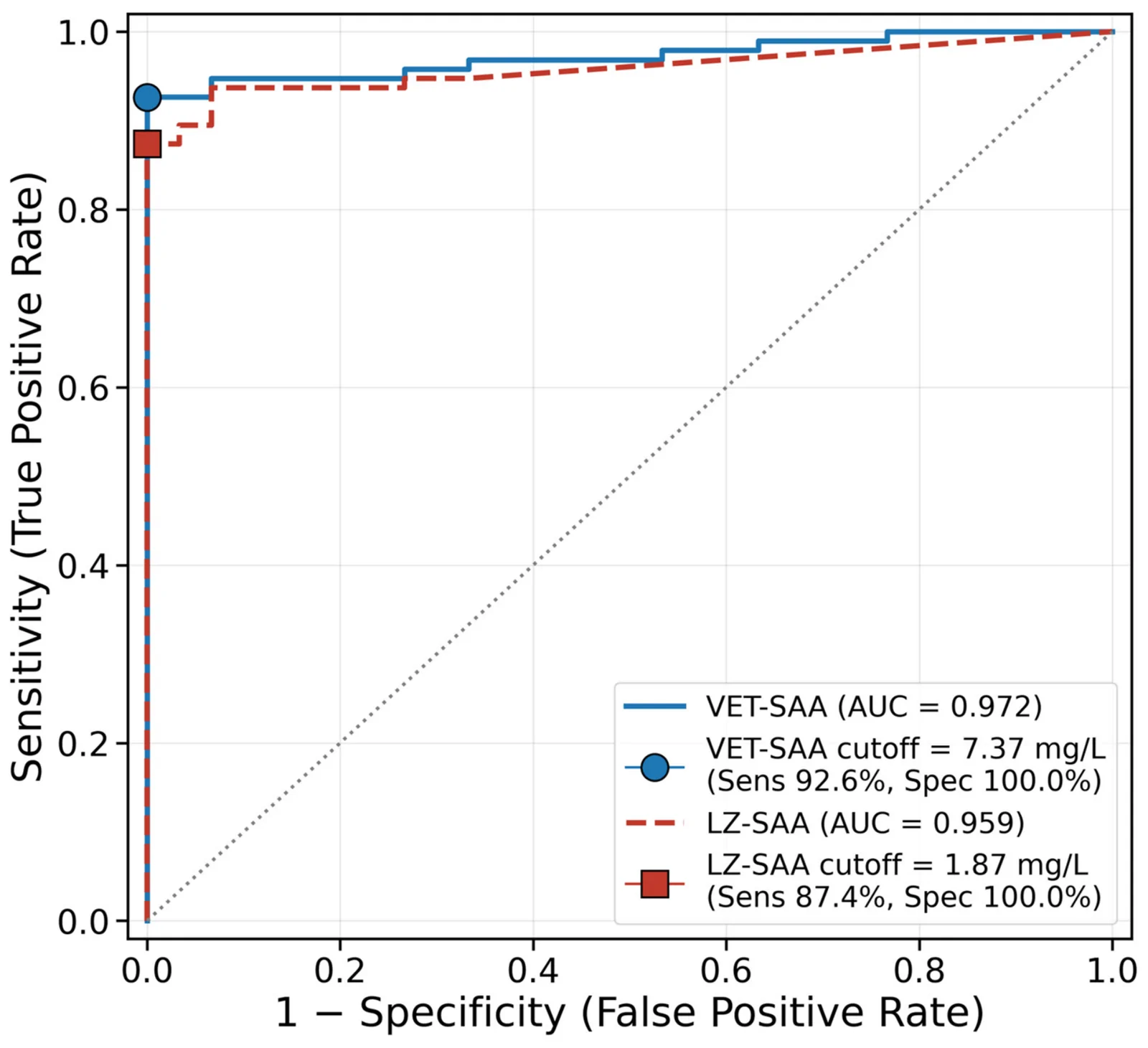
Receiver operating characteristic (ROC) curves for VET-SAA and LZ-SAA in Japanese sika deer (*Cervus nippon*). The optimal cutoff values determined by the Youden index are indicated by filled circles (VET-SAA) and squares (LZ-SAA). AUC, area under the curve; CI, confidence interval.

SAA concentrations were significantly higher in the diseased group than in the healthy group for both assays (Table 1; Figure 1). Of the 128 serum samples originally collected, 2 were excluded because of dilution-related assay issues and 1 was excluded as a likely duplicate measurement of another sample from the same individual and date, leaving 125 samples (30 healthy, 95 diseased) for the analyses described below. The median VET-SAA concentration was 2.66 mg/L (IQR: 1.88–4.29 mg/L) in the healthy group (n = 30) and 34.92 mg/L (IQR: 13.88–57.32 mg/L) in the diseased group (n = 95) (Mann–Whitney U test, *p* < 0.001). The median LZ-SAA concentration was 0.00 mg/L (IQR: 0.00–0.32 mg/L) in the healthy group and 89.34 mg/L (IQR: 7.39–167.56 mg/L) in the diseased group (*p* < 0.001). This difference remained highly significant after accounting for repeated sampling of some individuals using a linear mixed-effects model with individual identity as a random effect (log-transformed VET-SAA and LZ-SAA, both *p* < 0.001), confirming that the group difference was not an artifact of non-independent observations.

**Figure 2:**
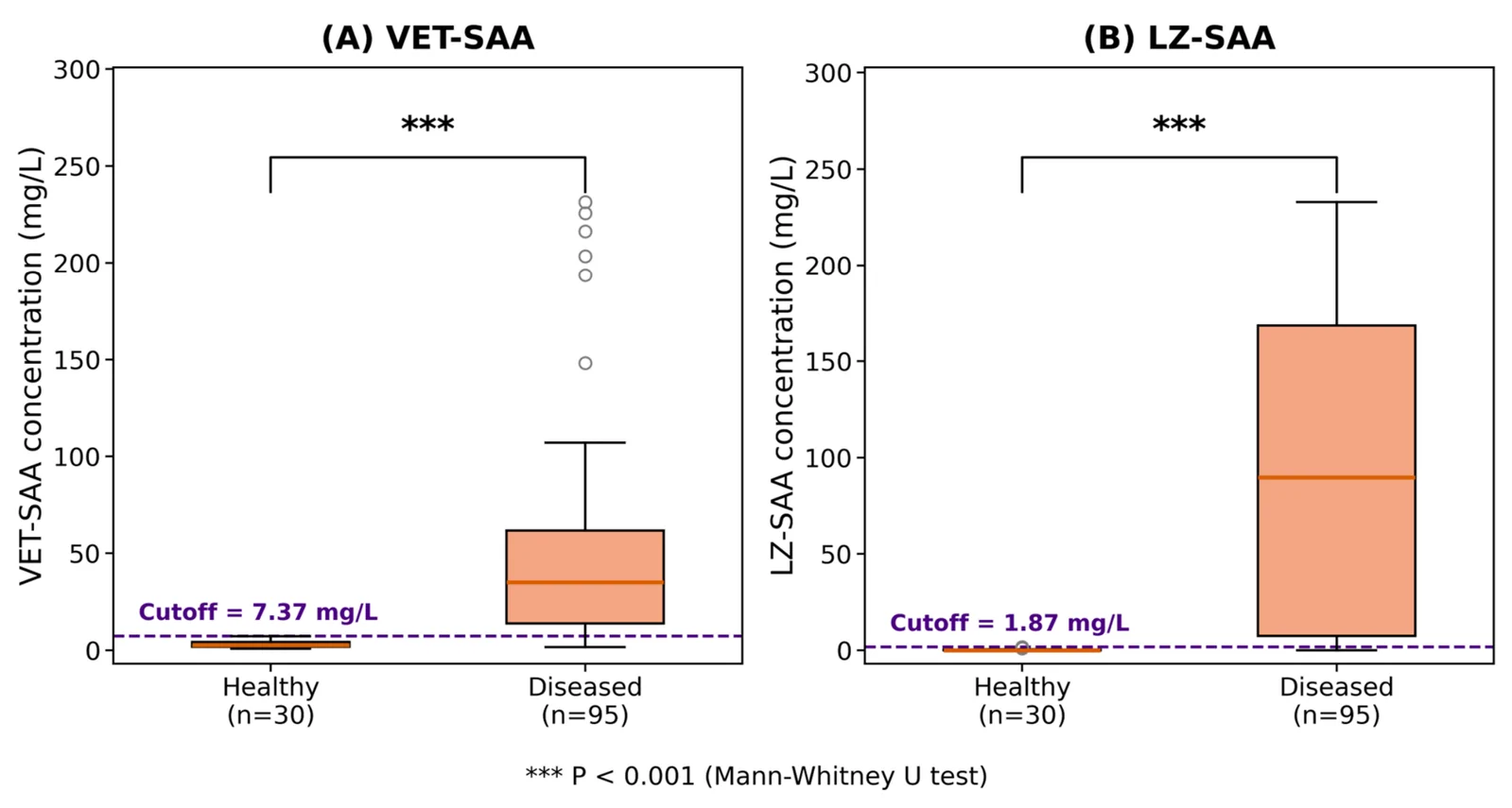
Comparison of SAA concentrations between healthy (n = 30) and diseased (n = 95) Japanese sika deer (*Cervus nippon*). (**A**) VET-SAA; (**B**) LZ-SAA. Boxes represent the interquartile range (IQR); horizontal lines within boxes indicate the median; whiskers extend to 1.5 × IQR; open circles represent outliers. Dashed lines indicate the optimal cutoff values (VET-SAA: 7.37 mg/L; LZ-SAA: 1.87 mg/L). *** *p* < 0.001 (Mann–Whitney U test).

ROC analysis revealed an AUC of 0.972 (95% CI: 0.941–0.993) for VET-SAA and 0.959 (95% CI: 0.924–0.986) for LZ-SAA (Figure 2). The optimal cutoff value was 7.37 mg/L for VET-SAA (sensitivity 92.6%, specificity 100.0%) and 1.87 mg/L for LZ-SAA (sensitivity 87.4%, specificity 100.0%). When the bootstrap was instead performed at the individual level to account for repeated sampling, the resulting confidence intervals were nearly identical (VET-SAA: 95% CI, 0.941–0.994; LZ-SAA: 95% CI, 0.923–0.987), indicating that the ROC-based diagnostic performance was robust to non-independence among repeated observations.

### 3.2. SAA Reference Intervals

Reference intervals for VET-SAA and LZ-SAA were calculated using serum samples from clinically healthy individuals (Table 2) [29]. For all animals combined, VET-SAA values were mean ± SD = 3.14 ± 1.80 mg/L (reference interval: 0.00–6.73 mg/L), and LZ-SAA values were 0.20 ± 0.38 mg/L (reference interval: 0.00–0.96 mg/L). Subspecies-specific VET-SAA reference intervals were 0.15–6.65 mg/L for Formosan sika deer (Hanajika), 0.00–6.32 mg/L for Mageshima sika deer, and 0.08–7.62 mg/L for mixed-subspecies individuals. LZ-SAA reference intervals were 0.00–1.50 mg/L, 0.00–0.34 mg/L, and 0.00–0.55 mg/L, respectively. In Mageshima sika deer and mixed-subspecies individuals, many LZ-SAA values were 0.00 mg/L, indicating a tendency for values to cluster in the low-concentration range. Within the diseased group, SAA concentrations also differed significantly among subspecies (Kruskal–Wallis test; VET-SAA: H = 6.57, *p* = 0.038; LZ-SAA: H = 6.53, *p* = 0.038). Median (range) VET-SAA concentrations in diseased animals were 35.41 (1.70–216.27) mg/L in Formosan sika deer (n = 54), 48.89 (6.32–231.41) mg/L in Mageshima sika deer (n = 28), and 15.76 (2.57–225.65) mg/L in mixed-subspecies individuals (n = 13); corresponding LZ-SAA values were 87.62 (0.00–232.84), 152.84 (0.34–214.86), and 9.73 (0.00–182.18) mg/L, respectively.

### 3.3. Correlations Between SAA and Serum Biochemical Parameters

**Figure 3:**
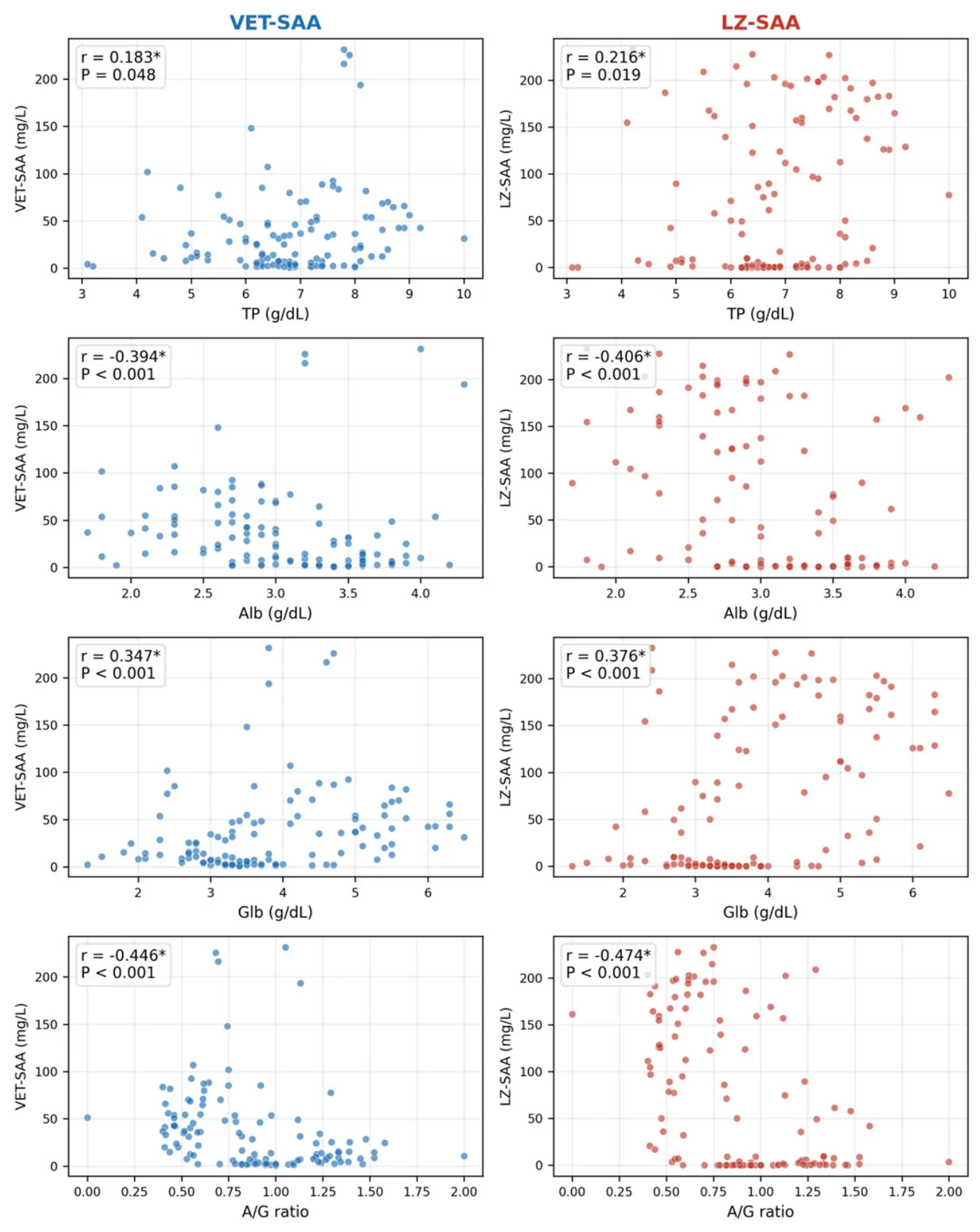
Scatter plots showing Spearman rank correlations between SAA concentrations and serum biochemical parameters in Japanese sika deer (*Cervus nippon*; n = 125). Left column: VET-SAA; right column: LZ-SAA. r, Spearman correlation coefficient; *p*, significance level. * *p* < 0.05, statistically significant.

Results of Spearman rank correlation analyses between SAA concentrations and serum biochemical parameters are shown in Table 3 and Figure 3. Both VET-SAA and LZ-SAA were significantly positively correlated with TP (VET-SAA: r = 0.183, *p* = 0.048; LZ-SAA: r = 0.216, *p* = 0.019) and Glb (VET-SAA: r = 0.347, *p* < 0.001; LZ-SAA: r = 0.376, *p* < 0.001). Conversely, both assays were significantly negatively correlated with Alb (VET-SAA: r = −0.394, *p* < 0.001; LZ-SAA: r = −0.406, *p* < 0.001) and the A/G ratio (VET-SAA: r = −0.446, *p* < 0.001; LZ-SAA: r = −0.474, *p* < 0.001). No significant correlation was observed with PLT for either assay. After accounting for repeated sampling using rmcorr (Table 4), the correlations with TP, Glb, and Alb (LZ-SAA only) remained significant, whereas the correlation between VET-SAA and Alb (*p* = 0.079) and between the A/G ratio and either assay (VET-SAA: *p* = 0.112; LZ-SAA: *p* = 0.060) were no longer statistically significant, indicating that these particular associations were less robust once individual-level non-independence was taken into account.

### 3.4. Correlations Between SAA and Hematological Parameters

**Figure 4:**
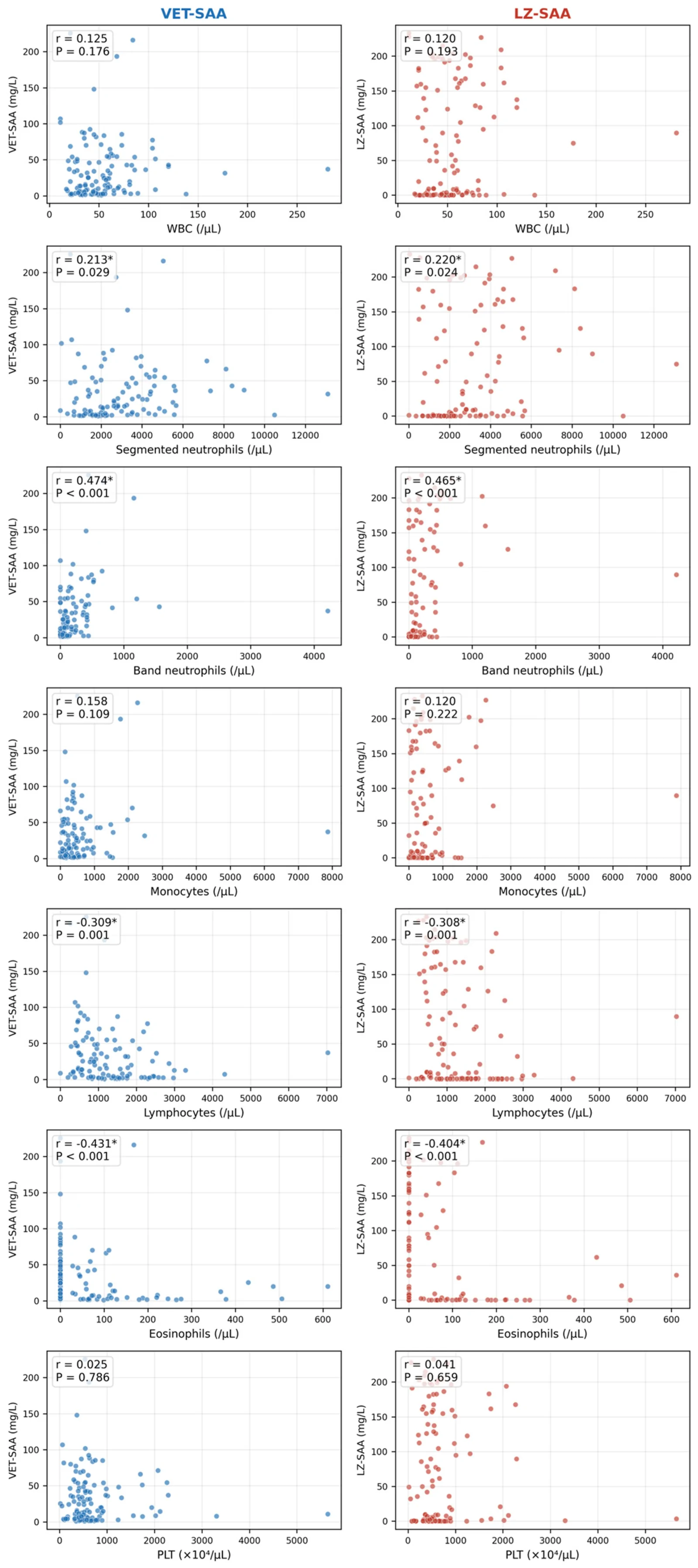
Scatter plots showing Spearman rank correlations between SAA concentrations and hematological parameters in Japanese sika deer (*Cervus nippon*; n = 125). Left column: VET-SAA; right column: LZ-SAA. r, Spearman correlation coefficient; *p*, significance level. * *p* < 0.05, statistically significant.

Results of Spearman rank correlation analyses between SAA concentrations and hematological parameters are shown in Table 3 and Figure 4. Both assays showed significant positive correlations with band neutrophils (VET-SAA: r = 0.474, *p* < 0.001; LZ-SAA: r = 0.465, *p* < 0.001). Significant negative correlations were observed with lymphocytes (VET-SAA: r = −0.309, *p* = 0.001; LZ-SAA: r = −0.308, *p* = 0.001) and eosinophils (VET-SAA: r = −0.431, *p* < 0.001; LZ-SAA: r = −0.404, *p* < 0.001).

A significant positive correlation with segmented neutrophils was observed for both assays (LZ-SAA: r = 0.220, *p* = 0.024; VET-SAA: r = 0.213, *p* = 0.029). No significant correlations were found between either assay and WBC or monocytes. However, after adjustment for repeated sampling using rmcorr (Table 4), the correlation with segmented neutrophils was no longer significant for either assay (VET-SAA: *p* = 0.351; LZ-SAA: *p* = 0.249), whereas the correlations with band neutrophils, lymphocytes, and eosinophils remained significant, indicating that the segmented neutrophil association was less robust than the other hematological correlations identified in this study.

## 4. Discussion

### 4.1. Measurement Characteristics of VET-SAA and LZ-SAA

In this study, we evaluated two SAA measurement systems—VET-SAA and LZ-SAA—using serum samples from Japanese sika deer. Both assays demonstrated similar correlation patterns with hematological and serum biochemical parameters, suggesting that both are applicable for inflammatory assessment in this species. Although both VET-SAA and LZ-SAA are LATIA reagents manufactured by Eiken Chemical Co., Ltd., VET-SAA was developed primarily for SAA measurement in domestic livestock, whereas LZ-SAA is a human SAA assay reagent. As previously reported in a bottlenose dolphin (*Tursiops truncatus*) with rhabdomyolysis, LZ-SAA may show lower sensitivity compared with species-specific assays in non-human animal species [16,22], suggesting that the cross-reactivity of LZ-SAA with Japanese sika deer SAA may be limited. Nevertheless, LZ-SAA achieved an AUC (0.959) comparable to that of VET-SAA (0.972). This apparent discrepancy can be explained by the fact that AUC reflects the rank-based discrimination between healthy and diseased groups across all possible thresholds, rather than the absolute analytical sensitivity of the assay. Because most healthy individuals showed LZ-SAA concentrations at or near the lower detection limit (median, 0.00 mg/L) while diseased individuals showed a marked and consistent elevation, the relative separation between the two groups remained large even though the absolute signal magnitude was lower than that of VET-SAA; this preserved the assay’s discriminative performance despite its reduced sensitivity at the low end of the concentration range.

This reduced sensitivity of LZ-SAA was also reflected in the dilution linearity and precision data. Across the full two-fold dilution series (1:1–1:256), the relationship between observed and expected LZ-SAA concentrations showed poor linearity (pooled R^2^ = 0.71 for the three samples tested), and this did not improve when the analysis was restricted to the lower dilution range (1:1–1:32) where recovery would be expected to be closer to proportional (pooled R^2^ = 0.64), indicating that the assay’s departure from the expected concentration is not confined to the highest dilutions alone. This finding is consistent with the intra-assay precision data, in which the coefficient of variation for LZ-SAA increased from 1.42 to 1.74% at high and medium concentrations to 51.06% at the low concentration tested (mean, 0.25 mg/L), indicating that LZ-SAA measurements become markedly imprecise as concentrations approach the lower end of the assay’s working range. Because many samples in this study, particularly from healthy animals, fell within or below this low-precision range, the combination of poor dilution linearity and high imprecision at low concentrations offers a plausible analytical explanation for the reduced sensitivity of LZ-SAA relative to VET-SAA observed in this species.

Both VET-SAA and LZ-SAA also showed a significant positive correlation with segmented neutrophils in this study. Segmented neutrophils are mature neutrophils mobilized from the bone marrow during inflammation and, like band neutrophils, are widely used as an indicator of inflammatory progression [30]. The fact that both assays shared this correlation with segmented neutrophils supports the conclusion that VET-SAA and LZ-SAA are both able to sensitively capture inflammation-associated neutrophil dynamics in Japanese sika deer. However, this correlation with segmented neutrophils did not remain statistically significant after adjustment for repeated sampling using rmcorr (Section 3.4; Table 4), in contrast to the correlation with band neutrophils, which remained significant in the rmcorr-adjusted analysis; the segmented neutrophil finding should therefore be interpreted with caution.

Comparison of SAA concentrations between the healthy and diseased groups revealed that both assays were significantly elevated in the diseased group (*p* < 0.001), supporting the utility of both assays for detecting inflammation in Japanese sika deer. The AUC values were 0.972 for VET-SAA and 0.959 for LZ-SAA, indicating excellent discriminatory ability. The optimal cutoff values were 7.37 mg/L for VET-SAA (sensitivity 92.6%, specificity 100.0%) and 1.87 mg/L for LZ-SAA (sensitivity 87.4%, specificity 100.0%). These cutoff values are consistent with the upper limits of the reference intervals established in this study (VET-SAA 6.73 mg/L; LZ-SAA 0.96 mg/L) and may serve as useful diagnostic references in clinical practice [29]. However, because the healthy group was defined strictly by the absence of any hematological or serum biochemical abnormality, it comprised a relatively small number of samples (n = 30); validation of these cutoff values in larger prospective studies with a larger healthy reference population is warranted.

### 4.2. Interpretation of Correlations Between SAA and Hematological Parameters

Both VET-SAA and LZ-SAA showed significant positive correlations with band neutrophils. Band neutrophils are immature neutrophils released from the bone marrow in response to acute inflammatory demand and are widely recognized as indicators of active inflammation [31]. The significant positive correlations observed in this study support the conclusion that SAA functions as an APP reflecting inflammatory status in Japanese sika deer, consistent with previous reports in Japanese Black cattle in which SAA was shown to be a sensitive indicator of inflammation [6].

No significant correlation was observed between either SAA assay and total WBC. In herbivores, including Japanese sika deer, the lymphocyte pool is proportionally large relative to total leukocytes. Even when segmented neutrophils increase during inflammation, the proportional change in total WBC may therefore be relatively small, limiting the sensitivity of total WBC as an inflammatory indicator [32,33,34]. The distinct kinetics of individual leukocyte subsets in this species likely contribute to the absence of a meaningful correlation between total WBC and SAA.

Both assays showed significant negative correlations with lymphocytes and eosinophils. Decreases in lymphocyte and eosinophil counts are well-recognized features of glucocorticoid-mediated stress responses, and these changes are more appropriately interpreted as reflecting a negative correlation with the stress response pattern rather than direct inflammatory effects [35,36,37]. Under inflammatory or stress conditions associated with elevated SAA concentrations, glucocorticoids induce lymphopenia and eosinopenia while increasing neutrophil counts, generating a characteristic stress leukogram. The findings of this study are consistent with this pattern, suggesting that SAA comprehensively reflects both inflammatory and stress responses in Japanese sika deer.

No significant correlation was found between either assay or monocytes. Monocytes are known to increase more prominently in chronic than in acute inflammation [30], and the absence of a correlation with the APP SAA is biologically plausible. These findings suggest that monocytes may have limited reliability as an inflammatory marker in Japanese sika deer, regardless of whether inflammation is acute or chronic.

### 4.3. Interpretation of Correlations Between SAA and Serum Biochemical Parameters

Both VET-SAA and LZ-SAA showed significant positive correlations with TP and Glb, and significant negative correlations with Alb and the A/G ratio. During inflammation, Glb fractions including APPs and immunoglobulins increase, while Alb decreases as a negative APP. The positive correlations with TP and Glb and negative correlations with Alb and the A/G ratio are therefore consistent with SAA reflecting inflammation-associated changes in serum proteins in Japanese sika deer.

In Japanese Black cattle, SAA has been reported to be significantly positively correlated with α-globulin, sialic acid, and fibrinogen, supporting its utility as a sensitive inflammatory indicator [6]. In the present study, total Glb rather than protein fractions was evaluated; however, since Glb encompasses APPs including SAA itself as well as immunoglobulins, the positive correlation between SAA and Glb is biologically plausible. Alb is a well-characterized negative APP that decreases during inflammation, and the negative correlations of Alb and A/G ratio with SAA are consistent with this property. Although Glb and A/G ratio are more commonly altered in chronic inflammatory conditions, sustained chronic inflammation is also associated with continued SAA elevation, which likely explains the significant correlations observed with these parameters. These findings suggest that SAA sensitively reflects protein changes associated with inflammatory states in Japanese sika deer. It should also be noted that TP, Alb, Glb, and the A/G ratio are not fully independent variables: Glb was calculated by subtracting Alb from TP, and the A/G ratio was calculated by dividing Alb by Glb; the correlations of SAA with these four parameters are therefore mathematically interrelated rather than fully independent findings. Furthermore, after adjustment for repeated sampling using rmcorr (Table 4), the correlation between VET-SAA and Alb (*p* = 0.079) and the correlations between the A/G ratio and both assays (VET-SAA: *p* = 0.112; LZ-SAA: *p* = 0.060) were no longer statistically significant, whereas the correlations with TP and Glb remained significant for both assays; the associations involving Alb and the A/G ratio should therefore be interpreted with more caution than those involving TP and Glb.

It should also be noted that TP, Alb, Glb, and the A/G ratio are not fully independent variables. Because Glb was calculated by subtracting albumin from total protein, and the A/G ratio was calculated as Alb divided by Glb, the correlations observed among these parameters are, in part, a mathematical consequence of their derivation rather than purely reflecting independent biological relationships. This interdependency should be considered when interpreting the correlation results presented above.

### 4.4. Clinical Application of SAA in Japanese Sika Deer

This study established reference intervals for SAA in Japanese sika deer and demonstrated significant associations between SAA and band neutrophils, TP, and Glb that remained robust after adjustment for repeated sampling, together with additional associations involving segmented neutrophils, Alb, and the A/G ratio that were less consistent once repeated sampling was accounted for (Section 3.3 and Section 3.4; Table 4). In zoological and wildlife medicine, early detection of inflammation before clinical signs become apparent is clinically important. Inflammatory assessment in Japanese sika deer has largely depended on WBC count, WBC differential, and serum protein changes, and reports on specific inflammatory markers remain scarce. The results of this study suggest that SAA could complement these conventional parameters as an inflammatory marker in this species [20,21]. Because the diseased group in this study encompassed a wide range of clinical conditions—including not only presumed infectious or inflammatory processes but also non-infectious causes such as traumatic injuries from intraspecific aggression, neurologic signs, dehydration, and abdominal disorders—the elevated SAA concentrations observed should be interpreted as reflecting a generalized inflammatory or illness response, whether of infectious or non-infectious origin, rather than any disease-specific pattern. The significant subspecies difference observed in SAA concentrations within the diseased group should also be interpreted cautiously, as it may reflect differences in the underlying distribution of disease conditions and severity among subspecies rather than an intrinsic subspecies effect on SAA production, particularly given the small and uneven sample sizes for some subspecies.

Both VET-SAA and LZ-SAA appear applicable for inflammatory assessment in Japanese sika deer. In particular, VET-SAA, which demonstrated a broader distribution of values and evident sensitivity to change in the low-concentration range compared with LZ-SAA, may be especially useful for detecting mild or early-stage inflammatory changes.

### 4.5. Limitations

This study has several limitations. First, because the healthy group was defined strictly by the absence of any hematological or serum biochemical abnormality in addition to the absence of overt clinical signs, the healthy group was limited to 30 samples, while the diseased group (n = 95) encompassed a broad range of disease conditions with few samples per category. In particular, the ROC-derived cutoff values were both calculated and evaluated using the same dataset, without an independent validation cohort; therefore, these cutoff values should be regarded as preliminary until confirmed in an external population. Validation with larger, more homogeneous populations is needed, particularly for the ROC-derived cutoff values and reference intervals.

Second, the study included multiple samples from the same individual (up to 8 samples per individual). Because samples collected at different time points were used to ensure an adequate sample size, the assumption of statistical independence was not fully met, and individual variation and within-individual variation could not be completely distinguished. Furthermore, as an exploratory study, correction for multiple comparisons was not applied; therefore, the possibility that some significant correlations arose by chance cannot be excluded, and correlations with *p*-values close to the 0.05 threshold (e.g., the correlation between VET-SAA and total protein, *p* = 0.048) should be interpreted with particular caution.

Third, this was a retrospective study using residual samples collected and stored previously, making it difficult to evaluate the clinical status and degree of inflammation in each individual using standardized criteria. Some samples were also missing WBC differential or other hematological data, limiting the number of samples available for certain analyses. Future prospective studies with larger, well-characterized populations are needed to further validate the reference intervals and cutoff values established in this study.

Fourth, because the number of animals available for this study was inherently limited by the size of the captive population at a single zoological facility, no a priori sample size calculation was performed; this constraint is common to reference interval studies in rare or non-domestic species, and the sample size used here (n = 84 individuals; 125 samples after exclusions) falls within the range considered acceptable for robust or non-parametric reference interval estimation under ASVCP guidelines when a minimum of 120 samples is not attainable [29]. Body weight was not available for most animals, as the majority of blood samples were collected without chemical restraint during routine veterinary care to minimize animal stress; body weight was recorded only opportunistically for the small subset of animals sampled under anesthesia (approximately 30–40 kg), which was considered insufficient to calculate a representative mean for the study population. In addition, because serum samples were accumulated and measured over an extended period rather than in a single analytical batch, inter-assay variability between measurement occasions cannot be excluded, although intra-assay reproducibility is expected to conform to the manufacturer’s specifications.

Fifth, the reference intervals in this study were calculated using the mean ± 2 SD method with the lower limit truncated at zero when negative, following common practice in veterinary reference interval studies. Formal evaluation of the healthy-group data (Shapiro–Wilk test) indicated that VET-SAA concentrations did not conform to a normal distribution on the raw scale (*p* = 0.003) but approximated normality after log-transformation (*p* = 0.46), consistent with the typically log-normal distribution of biomarker concentrations; in contrast, LZ-SAA concentrations remained markedly non-normal even after log-transformation (*p* < 0.001), reflecting the large proportion of samples at or below the assay’s detection limit (17 of 30 healthy samples = 0.00 mg/L). No values were identified as statistical outliers by Tukey’s fence method (1.5 × interquartile range) for VET-SAA; for LZ-SAA, the two highest healthy-group values (1.04 and 1.72 mg/L, both in Formosan sika deer) fell outside Tukey’s fences, but these values remained within the assay’s normal working range and were not excluded, as this pattern likely reflects the zero-inflated distribution of LZ-SAA rather than a true outlier. Given the non-normal distribution of both assays, particularly LZ-SAA, and the small subspecies-specific sample sizes (Formosan sika deer, n = 11; Mageshima sika deer, n = 13; mixed-subspecies individuals, n = 6), which fall below the minimum of 20 samples recommended by ASVCP guidelines even for robust reference interval estimation [29], the subspecies-specific reference intervals reported in Table 2 should be interpreted as preliminary reference limits rather than fully validated reference intervals, and should be confirmed in larger, subspecies-specific cohorts in future studies. The overall reference interval (n = 30, combining all subspecies) approaches, but remains below, the minimum sample size generally recommended for robust reference interval estimation, and should likewise be interpreted with appropriate caution.

## 5. Conclusions

In this study, SAA concentrations were measured in serum samples from captive Japanese sika deer using VET-SAA and LZ-SAA assays. Reference intervals were established, and significant correlations were identified between SAA and band neutrophils, TP, and Glb that were robust to adjustment for repeated sampling, along with additional associations involving segmented neutrophils, Alb, and the A/G ratio that were less consistent after this adjustment. SAA concentrations were significantly higher in diseased individuals than in healthy individuals for both assays, and ROC analysis demonstrated excellent discriminatory ability (AUC: 0.972 for VET-SAA, 0.959 for LZ-SAA). These findings support the conclusion that SAA functions as an APP reflecting inflammatory status in Japanese sika deer. Both VET-SAA and LZ-SAA are applicable for inflammatory assessment in this species, with VET-SAA potentially offering advantages for detecting mild or early-stage inflammatory changes.

## Figures and Tables

**Table 1:** Comparison of SAA concentrations between healthy and diseased Japanese sika deer, and results of ROC analysis for discriminating diseased from healthy individuals.

		n	VET-SAA (mg/L), Median (IQR)	LZ-SAA (mg/L), Median (IQR)
Healthy		30	2.66 (1.88–4.29)	0.00 (0.00–0.32)
Diseased		95	34.92 (13.88–57.32)	89.34 (7.39–167.56)
*p* value a			<0.001	<0.001
**Assay**	**AUC (95% CI)**	**Optimal cutoff (mg/L) b**	**Sensitivity (%)**	**Specificity (%)**
VET-SAA	0.972 (0.941–0.993)	7.37	92.6	100.0
LZ-SAA	0.959 (0.924–0.986)	1.87	87.4	100.0

*a Mann–Whitney U test. b Optimal cutoff determined as the point maximizing the Youden index (sensitivity + specificity − 1). Healthy group (n = 30) was strictly defined as animals with no abnormality in clinical findings or hematological/serum biochemical parameters; the diseased group (n = 95) comprised all remaining individuals. Bold indicates the overall total row.*

**Table 2:** Reference intervals of VET-SAA and LZ-SAA concentrations in clinically healthy sika deer (*Cervus nippon*) by subspecies.

Subspecies	n	VET-SAA (mg/L)			LZ-SAA (mg/L)		
		Mean ± SD	Range	Reference Interval a	Mean ± SD	Range	Reference Interval a
**Hanajika (C. n. taioanus)**	11	3.40 ± 1.63	1.58–6.14	0.15–6.65	0.41 ± 0.55	0.00–1.72	0.00–1.50
**Mageshika (C. n. mageshimae)**	13	2.59 ± 1.86	0.89–7.01	0.00–6.32	0.07 ± 0.13	0.00–0.42	0.00–0.34
**Mixed subspecies b**	6	3.85 ± 1.89	2.04–7.23	0.08–7.62	0.09 ± 0.23	0.00–0.56	0.00–0.55
**Total**	**30**	**3.14 ± 1.80**	**0.89–7.23**	**0.00–6.73**	**0.20 ± 0.38**	**0.00–1.72**	**0.00–0.96**

*a Reference interval calculated as mean ± 2 SD (lower limit set to 0 where negative). b Individuals of mixed subspecies origin, tentatively considered hybrids involving the Honshu sika deer (C. n. nippon) and/or Kyushu-type sika deer, based on the zoo’s animal records; the exact subspecies composition has not been genetically confirmed. Bold indicates the combined total across all subspecies.*

**Table 3:** Correlation coefficient analysis of LZ- and VET-SAA with blood test parameters (n = 125; excludes 2 dilution-affected samples and 1 duplicate sample).

	WBC	Seg	Band	Mon	Lym	Eos	PLT	TP	Alb	Glob	A/G
LZ-SAA											
Correlation coefficient	0.120	0.220	0.465	0.120	−0.308	−0.404	0.041	0.216	−0.406	0.376	−0.474
*p*-value	0.193	0.024 *	<0.001 *	0.222	0.001 *	<0.001 *	0.659	0.019 *	<0.001 *	<0.001 *	<0.001 *
VET-SAA											
Correlation coefficient	0.125	0.213	0.474	0.158	−0.309	−0.431	0.025	0.183	−0.394	0.347	−0.446
*p*-value	0.176	0.029 *	<0.001 *	0.109	0.001 *	<0.001 *	0.786	0.048 *	<0.001 *	<0.001 *	<0.001 *

WBC, leukocyte count; Seg, segmented neutrophil count; Band, band neutrophil count; Mon, monocyte count; Lym, lymphocyte count; Eos, eosinophil count; PLT, platelet count; TP, total protein; Alb, albumin; Glob, globulin; A/G, albumin/globulin ratio. * p < 0.05, statistically significant with Spearman’s correlation coefficient analysis.

**Table 4:** Repeated measures correlation (rmcorr) analysis of LZ- and VET-SAA (log-transformed) with blood test parameters, accounting for non-independence of repeated samples from the same individual (n = 125 samples) [27].

	WBC	Seg	Band	Mon	Lym	Eos	PLT	TP	Alb	Glob	A/G
LZ-SAA											
Correlation coefficient	0.293	0.214	0.363	0.276	−0.473	−0.474	0.109	0.361	−0.331	0.436	−0.308
*p*-value	0.079	0.249	0.045 *	0.132	0.007 *	0.009 *	0.514	0.026 *	0.045 *	0.006 *	0.060
VET-SAA											
Correlation coefficient	0.203	0.173	0.413	0.353	−0.487	−0.405	0.135	0.326	−0.292	0.373	−0.262
*p*-value	0.228	0.351	0.021 *	0.051	0.005 *	0.029 *	0.420	0.046 *	0.079	0.021 *	0.112

Abbreviations as in Table 3. * p < 0.05, statistically significant.

## Data Availability

The complete raw dataset is not publicly deposited because the underlying animal records are maintained by Kagoshima City Hirakawa Zoological Park as part of its institutional records; additional data beyond the minimal dataset are available from the corresponding author upon reasonable request.
